# Quantifying Tumor Heterogeneity from Multiparametric Magnetic Resonance Imaging of Prostate Using Texture Analysis

**DOI:** 10.3390/cancers14071631

**Published:** 2022-03-23

**Authors:** Saleh T. Alanezi, Frank Sullivan, Christoph Kleefeld, John F. Greally, Marcin J. Kraśny, Peter Woulfe, Declan Sheppard, Niall Colgan

**Affiliations:** 1 Physics Department, Faculty of Science, Northern Border University, Arar 1321, Saudi Arabia; 2 School of Physics, College of Science and Engineering, National University of Ireland Galway, H91 CF50 Galway, Ireland; christoph.kleefeld@nuigalway.ie (C.K.); m.j.krasny@nuigalway.ie (M.J.K.); niall.colgan@nuigalway.ie (N.C.); 3 Prostate Cancer Institute, Department of Radiation Oncology, Galway Clinic, H91 HHT0 Galway, Ireland; frank.sullivan@galwayclinic.com; 4 School of Medicine, College of Medicine, Nursing, National University of Ireland Galway, H91 TK33 Galway, Ireland; 5 Department of Pathology, Galway Clinic, H91 HHT0 Galway, Ireland; john.greally@yahoo.com; 6 Department of Radiology, Galway Clinic, H91 HHT0 Galway, Ireland; peter.woulfe@galwayclinic.com (P.W.); declan.sheppard@hse.ie (D.S.); 7 Department of Radiology, University Hospital Galway, H91 YR71 Galway, Ireland

**Keywords:** texture analysis, prostate cancer, prostatectomy, multiparametric MRI (mp-MRI), heterogeneity

## Abstract

**Simple Summary:**

Prostate cancer (PCa) occurs in males at a rate of 21.8%, predominantly at the customary primary site. High cure rates are possible through early detection and therapy when the tumor is still restricted to the prostate. These tumors do not grow rapidly, allowing for periods of up to 20 years between diagnosis and death. Multiparametric MRI (mp-MRI) is used as a non-invasive approach to diagnose PCa in subjects. This imaging method uses MR imaging with at least one functional MRI sequence to detect and characterize PCa. The use of multiparametric magnetic resonance imaging has refined the diagnosis of prostate cancer in radiology. Malignancy-modified critical features in tissue composition, such as heterogeneity, are associated with adverse tumor biology. Heterogeneity can be quantified through texture analysis, an effective technique for reviewing tumor images acquired in routine clinical practice. This study focused on identifying and quantifying tumor heterogeneity from prostate mp-MRI utilizing texture analysis.

**Abstract:**

(1) Background: Multiparametric MRI (mp-MRI) is used to manage patients with PCa. Tumor identification via irregular sampling or biopsy is problematic and does not allow the comprehensive detection of the phenotypic and genetic alterations in a tumor. A non-invasive technique to clinically assess tumor heterogeneity is also in demand. We aimed to identify tumor heterogeneity from multiparametric magnetic resonance images using texture analysis (TA). (2) Methods: Eighteen patients with prostate cancer underwent mp-MRI scans before prostatectomy. A single radiologist matched the histopathology report to single axial slices that best depicted tumor and non-tumor regions to generate regions of interest (ROIs). First-order statistics based on the histogram analysis, including skewness, kurtosis, and entropy, were used to quantify tumor heterogeneity. We compared non-tumor regions with significant tumors, employing the two-tailed Mann–Whitney U test. Analysis of the area under the receiver operating characteristic curve (ROC-AUC) was used to determine diagnostic accuracy. (3) Results: ADC skewness for a 6 × 6 px filter was significantly lower with an ROC-AUC of 0.82 (*p* = 0.001). The skewness of the ADC for a 9 × 9 px filter had the second-highest result, with an ROC-AUC of 0.66; however, this was not statistically significant (*p* = 0.08). Furthermore, there were no substantial distinctions between pixel filter size groups from the histogram analysis, including entropy and kurtosis. (4) Conclusions: For all filter sizes, there was poor performance in terms of entropy and kurtosis histogram analyses for cancer diagnosis. Significant prostate cancer may be distinguished using a textural feature derived from ADC skewness with a 6 × 6 px filter size.

## 1. Introduction

Prostate cancer is the second most frequent tumor identified in men worldwide and the leading cause of mortality in this population [1]. The prostate is a male reproductive organ with the diameter of a walnut that produces an alkaline fluid comprising about 20–30% of the ejaculate [2]. The use of multiparametric magnetic resonance imaging (mp-MRI) has improved the clinical diagnosis of prostate carcinoma [3]. mp-MRIs normally encompass T_1_ and T_2_ anatomical sequences and diffusion-weighted imaging (DWI), and generate the apparent diffusion coefficient (ADC) for the prostate [4,5].

Malignancies are heterogeneous at the genetic and histopathological levels, having dimensional alterations in cellularity, angiogenesis, extravascular and extracellular matrices, and regions of necrosis [6,7,8]. Tumor heterogeneity is a good metric to identify therapy sensitivity and possible therapeutic efficacy. Lesions with severe heterogeneity are associated with poor outcomes [9]. Tumor heterogeneity is difficult to assess from either irregular sampling or biopsy. Establishing a non-invasive technique to assess the heterogeneity of a tumor is a clinical necessity [10,11].

TA is an image processing method that quantifies heterogeneity depending on the spatial dissemination of pixel levels potentially missed by the naked eye [5]. Imaging provides quantifiable, clinically relevant tumor parameters for assessing heterogeneity. Applying image signal heterogeneity and pattern recognition in the analysis of differences that are undetectable by the human eye to regular clinical imaging procedures may greatly enhance and aid diagnosis [8]. TA uses several mathematical methods to assess an object’s gray level strength and pixel positions to produce texture attributes indicating intralesional heterogeneity [8,12,13]. Statistically based techniques are commonly used to describe gray-level value distribution and relationships. In statistically built TA, two parameter classes are described: first- and second-order statistical methods. The first-order correlates gray-level frequency division in the area of interest from the histogram of pixel intensities [8,14]. TA has been successfully applied to oncology studies on tumor detection, grading, diagnosis, and reviews of treatment response [8,12,15]. TA provides additional heterogeneity metrics for tissue that may increase accuracy when differentiating tumor tissue from adjacent benign nodular tissue [13,15]

The use of enhanced radical prostatectomy (RP) data as input has improved the classification of TA metrics utilized in prostate lesion identification [16,17]. A pathologist’s appropriate investigation of the RPs is significant in deciding the necessity for additional therapy and the prognosis of the subject. Standardization of the procedures used for tissue specimen mounting and reporting has provided consistent and comprehensive data and clinical reporting [18]. Histopathological assessments provide the gold standard for microscopic heterogeneity. Radical prostatectomy decisions depend on histological features, including extraprostatic extension, Gleason grade, tumor identification, and spreading toward the seminal vesicles [18,19,20,21,22].

In the present study, we aimed to quantify prostate tumors and assess carcinoma heterogeneity from prostate multiparametric magnetic resonance images using texture analysis.

## 2. Materials and Methods

### 2.1. Patient Group

**Figure 1:**
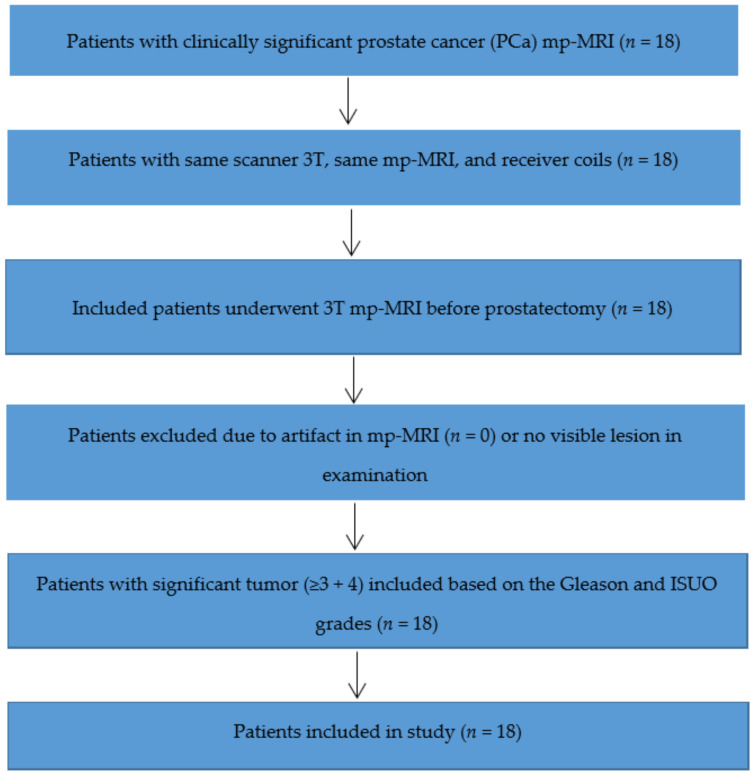
Flowchart of inclusion and exclusion criteria for patient selection.

The group contained 18 patients with suspected prostate malignancies who received a prostatic mp-MRI prior to diagnosis and were undergoing prostatectomy. Men with a tumor in the transition zone (TZ) or peripheral zone (PZ) underwent a biopsy before mp-MRI and were diagnosed based on systemic biopsy. In the study population, the maximum time interval was 1 month from biopsy to mp-MRI and 3 months from prostatectomy to mp-MRI. The exclusion and inclusion criteria (shown in Figure 1) were: (a) patients scanned in the same scanner 3T with the same mp-MRI and receiver coils were included; (b) patients who underwent 3T mp-MRI before prostatectomy were included; (c) patients were excluded if their mp-MRI and examination indicated an artifact or no visible lesion; and (d) patients with a significant tumor (≥3 + 4) at radical prostatectomy were included. Table 1 outlines the demographics for 18 subjects with a mean age of 63 (55–76 years), a mean prostate-specific antigen (PSA) serum concentration of 7.5 ng/mL (ranging 1.2–10.9 ng/mL), and a mean tumor area of 1.58 cm^2^ (at histology post-prostatectomy). 

### 2.2. Multiparametric Magnetic Resonance Imaging

**Figure 2:**
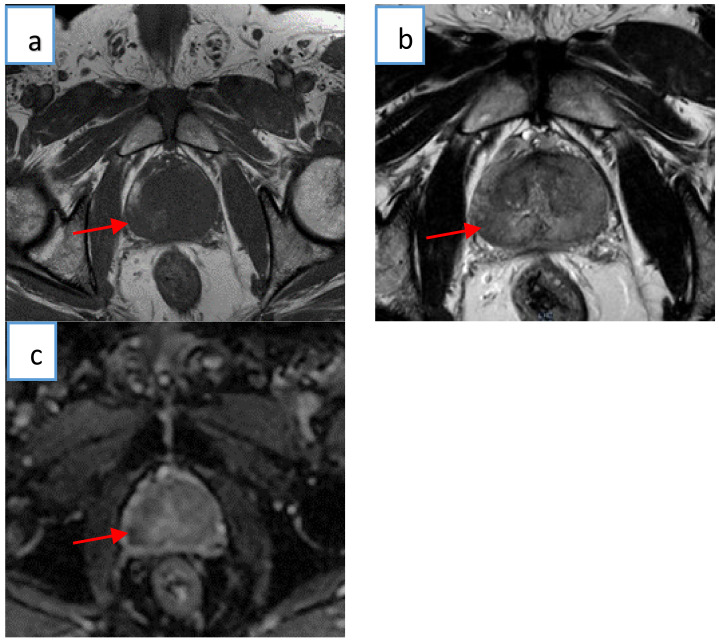
Significant tumor from a single axial slice (arrow images); (**a**) T_1_-weighted image; (**b**) T_2_-weighted image; (**c**) ADC map image. Images are from a 58-year-old patient with a significant tumor (≥3 + 4) according to the Gleason and ISUO grades.

Subjects underwent mp-MRI scans utilizing a Philips Ingenia 3T mp-MRI (Philips medical system) and receiver coils. The mp-MRI approach included the acquisition of a T_1_-weighted image, T_2_-weighted images, and an ADC map image. Apparent diffusion coefficient values were automatically measured by the software and presented as a parametric map reflecting the level of diffusion for water molecules. Table 2 illustrates the mp-MRI acquisition parameters in detail. Figure 2 presents mp-MRI images from a patient with a clinically verified prostate tumor.

### 2.3. Prostatectomy Specimen Procedure

The following is the procedure used to process prostatectomy specimens in the histopathology laboratory. The prostate is received in a fresh state, assigned a histopathology number, and photographed. The seminal vesicles are removed, and the prostate is then weighed [23] by placing the fresh prostate in a graduated cylindrical structure containing a precisely measured volume of water. The displacement level of the water is recorded proportionate to the volume of the gland (Archimedes’ principle) [24]. The gland and its separated seminal vesicles are placed in neutral buffered formalin for approximately 48 h [25]. Following this initial fixation, the right lobe of the prostate is painted with red ink, the left lobe with green ink, and black ink is applied over the apex and base [19]. The prostate is fixed for a further 30–60 min to allow the ink to fully adhere to the surface. The gland is rinsed with cold water and patted dry with tissues. The gland is then serially sectioned at 4 mm intervals. The serial sections are then laid out onto a blue background and assigned identifying labels, e.g., A1, A2, etc., from apex to base. Tissue sections are stained routinely with hematoxylin and eosin stains [21].

For the microscopic examination, macroslides are used for larger than usual sections obtained from prostate tissue blocks. These sections were systematically examined by one or two pathologists depending on the complexity of the case. The tissue section area was measured using a transparent plastic grid in which each square centimeter was divided into 25 smaller squares, measuring 0.04 cm^2^ individually. In this way, it was possible to obtain a closer approximation of the tumor’s true area. We confirmed the accuracy of our measurements using a digital system (Hitachi).

The report sent to the surgeon for each radical prostatectomy included a written account of the histopathology findings and a schematic representation of the tumor location, grade, quantity, and proportion (%) in each affected section. Pericapsular and perineural spread were illustrated, as well as seminal vesicle involvement. This illustrated report also included a schematic representation of pre-operative biopsy results compared with the radical prostatectomy findings. All cases were discussed with the urology surgeons at a multidisciplinary meeting.

### 2.4. Histopathology Review

Prostatectomy specimens were reported by a histopathologist specializing in the interpretation of prostate cancer at University Hospital Galway. Patients were labeled as having a significant tumor (≥3 + 4) based on the Gleason and ISUO grades or no significant cancer (≤3 + 4) [5,26].

### 2.5. Histology–MRI Matching

An experienced radiologist (D.S.), knowledgeable about histological findings, reviewed each dataset using Picture Archiving and Communication System (PACS) DICOM viewer and aligned the single axial slice focal most suspected cancer to the site of cancer proven according to histopathology report.

However, mp-MRI regions of interest (ROIs), including ADC, T_2_, and T_1_, were guided by a radiologist (D.S.). The most inclusive tumor diameter on a single axial slice was used to determine an ROI for each ADC, T_2_, and T_1_. Non-tumor regions were examined based on the same zone (in different regions) examined for each patient’s tumor.

### 2.6. Magnetic Resonance Textural Analysis

mp-MRI regions of interest (ROIs) were guided by an experienced radiologist, as described in Section 2.5. The ADC map, and T_2_- and T_1_-weighted ROIs from the decided axial slice showing a non-tumor region and a malignant tumor were processed. We then adjusted the focus for the most inclusive tumor diameter based on the histopathology reports for every patient. The images were subjected to mp-MRI texture analysis (mp-MRI TA) using in-house designed MATLAB software (v. 2019a, MathWorks, Natick, MA, USA) and verified using ImageJ (Rasband, W.S., ImageJ, U.S.A., National Institutes of Health, Bethesda, MA, USA, 1997–2018) and MaZda software [27]. mp-MRI TA consisted of image histogram interpretation followed by the quantification of textures. Firstly, the initial filtration step highlighted ROIs with texture attributes from a selection of specified filter scales (3 × 3, 6 × 6, and 9 × 9 px) using a fast Fourier transform-based bandpass filter in ImageJ (Joachim Walter’s FFT Filter). This step filtered out ROIs of the specified size using Gaussian filtering in the Fourier space, allowing a histogram interpretation of the refined image [13,28]. Secondly, image histogram interpretation was used to quantify tumor heterogeneity based on a first-order statistical analysis of skewness, kurtosis, and entropy. These specifications emulate the amount, intensity, and variation in areas of high and low signal intensity. The symmetry of the image intensity was determined by skewness. Entropy is a parameter evaluating the disorder of interpixel concentrations at the individual filter scale. Kurtosis determines the sharpness of the histogram dissemination [13,29]. 

### 2.7. First-Order Statistical Method

The attribute of gray-level distribution is determined for an image area based on its pixels, whereas its properties are quantified and exploited through space relations fundamental to the gray-level distribution [30]. When using this type of analysis, if the picture is a derivative *f*(*x*,*y*) of space parameters x and y, (*x* = 0, 1, …, *N* − 1 and *y* = 0, 1, …, *M* − 1), then *f*(*x*,*y*) has distinct values *i* = 0, 1, …, *G* − 1, where *G* is the whole number of the image strength degree [29]. The strength degree histogram is a metric that exhibits the number of image pixels using the intensity as defined in Equations (1) and (2):

(1) $$h\left(i\right)=\sum_{x=0}^{N-1}\sum_{x=0}^{M-1}\delta \left(f\left(x,y\right),i\right)$$

where *δ*(*j*,*i*) is the Kronecker delta function:

(2) $$\delta \left(j,i\right)=\left\{\begin{array}{c} 1\;,\;j=i\\ 0,\;j\neq i \end{array}\right.$$

The intensity-level histogram effectively summarizes the statistical information of the image. The gray-level histogram includes first-order statistical information and comprises individual pixels. If the intensity degree histogram is distributed by the whole number of pixels, then the estimated possible concentration of intensity levels is obtained by Equation (3).

(3) $$P\left(i\right)=\frac{h\left(i\right)}{NM}\;i=0,\;1,\;\ldots \;,\;G-1$$

The histogram can be used to quantify the first-order statistical method of the image, thus providing image features. Furthermore, it can be used to characterize the texture using Equations (4)–(8) [29].

(4) $$Mean:\;µ=\sum_{i=0}^{G-1}ip\left(i\right)$$

(5) $$Variance:\;\sigma^{2}=\sum_{i=0}^{G-1}{\left(i-µ\right)}^{2}p\left(i\right)$$

(6) $$Skewness:\;{µ}_{3}=\sigma^{-3}\sum_{i=0}^{G-1}{\left(i-µ\right)}^{3}p\left(i\right)$$

(7) $$Kurtosis:\;{µ}_{4}=\sigma^{-4}\sum_{i=0}^{G-1}{\left(i-µ\right)}^{4}p\left(i\right)-3$$

(8) $$Entropy:\;H=-\sum_{i=0}^{G-1}p\left(i\right)\;log_{2}\left[p\left(i\right)\right]$$

### 2.8. Statistical Analysis

The attributes for malignant and nontumor ROIs were assessed for each textural metric using a two-tailed Mann–Whitney U test (*p*-value determined at *p* < 0.05). ROC interpretations represented textural features derived from the area of interest of every ADC and T_1_ and T_2_-weighted image to anticipate aggressive prostate cancer. The ROC-AUC and 95% confidence intervals were used to detect ideal individual parameters. The false discovery rate was maintained at less than 5% using the Benjamini–Hochberg procedure [31]. The statistical interpretations were completed using SPSS Statistics software (Windows version 26; IBM, Armonk, NY, USA).

## 3. Results

### 3.1. Patient Group

Malignant tumors ranged from 0.4 to 3.6 cm^2^ in size, with a mean size of 1.58 cm^2^. Table 3, Table 4 and Table 5 represent the measures of the ROIs’ MR textural variables for subjects with either non-tumor regions or lesion tumors. *p*-values indicate the significant difference between ROIs for each subject, including non-tumor regions and significant tumors. The textural parameters of the ROC-AUC values for mp-MRI were examined and used to distinguish significant and non-tumor regions in ROI images.

### 3.2. Textural Parameters

Table 3, Table 4 and Table 5 illustrate the textural measures for skewness, kurtosis, and entropy. Details for each parameter are explained below. 

Data represented by the mean ± SEM value, skewness of ADC (3 × 3, 6 × 6, and 9 × 9 px), T_2_ (3 × 3, 6 × 6, and 9 × 9 px), and T_1_ (3 × 3, 6 × 6, and 9 × 9 px) filter sizes were ADC (0.06 ± 0.12, −0.14 ± 0.13, −0.13 ± 0.12), T_2_ (0.28 ± 0.94, 0.14 ± 0.15, −0.03 ± 0.16), and T_1_ (−0.16 ± 0.78, 0.05 ± 0.13, −0.20 ± 0.17) for non-tumor regions, and ADC (0.43 ± 0.15, 0.49 ± 0.11, 0.17 ± 0.12), T_2_ (0.01 ± 0.10, 0.06 ± 0.03, 0.12 ± 0.10), and T_1_ (0.12 ± 0.10, 0.06 ± 0.17, −0.05 ± 0.45) for malignant tumors. The median skewness of the ADC (6 × 6 px) filter size was considerably less for subjects (*p* = 0.001), although median T_1_ and T_2_ levels of skewness for the remaining filter sizes were not statistically significant (Table 3).

Data represented by the mean ± SEM value, kurtosis of ADC (3 × 3, 6 × 6, and 9 × 9 px), T_2_ (3 × 3, 6 × 6, and 9 × 9 px), and T_1_ (3 × 3, 6 × 6, and 9 × 9 px) filter sizes were ADC (−0.72 ± 0.10, −0.64 ± 0.16, −0.78 ± 0.08), T_2_ (0.13 ± 0.11, −0.01 ± 0.24, −0.09 ± 0.19), and T_1_ (−0.57 ± 0.14, 0.01 ± 0.19, −0.08 ± −0.29) for non-tumor regions, and ADC (−0.41 ± 0.22, −0.47 ± 0.19, −0.65 ± 0.10), T_2_ (0.19 ± 0.15, −0.34 ± 0.16, −0.51 ± 0.14), and T_1_ (−0.05 ± 0.45, 0.22 ± 0.43, 0.22 ± 0.43) for malignant tumors. Kurtosis medians in reference to ADC, T_2_, and T_1_ (3 × 3, 6 × 6, and 9 × 9 px) filters were not significant for *p*-values of ADC (*p* = 0.37, 0.22, 0.81), T_2_ (*p* = 0.6, 0.72, 0.82), and T_1_ (*p* = 0.46, 0.65, 0.56) (Table 4).

Data represented by the mean ± SEM value, entropy of ADC (3 × 3, 6 × 6, and 9 × 9 px), T_2_ (3 × 3, 6 × 6, and 9 × 9 px), and T_1_ (3 × 3, 6 × 6, and 9 × 9 px) were ADC (4.09 ± 0.09, 4.08 ± 0.09, 4.02 ± 0.10), T_2_ (6.25 ± 0.11, 5.92 ± 0.12, 5.72 ± 0.13), and T_1_ (5.61 ± 0.30, 4.98 ± 0.31, 4.58 ± 0.27) for non-tumor regions, and were ADC (4.07 ± 0.15, 4.15 ± 0.11, 4.12 ± 0.11), T_2_ (6.10 ± 0.22, 5.86 ± 0.12, 5.59 ± 0.16), and T_1_ (5.48 ± 0.31, 4.85 ± 0.27, 4.60 ± 0.24) for malignant tumors. The entropy medians were not significantly different between tumors and non-tumor regions (*p* = 0.56–0.89) (Table 5).

### 3.3. Disease Classification (Textural Metrics)

**Figure 3:**
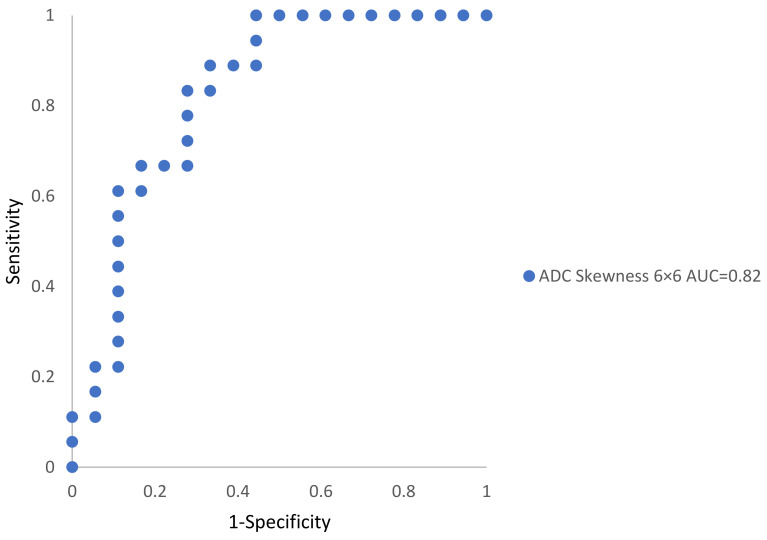
The best textural features from ROC curves and discernment of ROIs including significant tumors or non-tumor regions with AUC value.

ADC skewness for a 6 × 6 px filter was the only reliable acting classifier with a ROC-AUC of 0.82 (95% CI). Figure 3 shows the ROC-AUCs for significant univariate metrics that manifested the reinforced specificity and sensitivity.

## 4. Discussion

We used mp-MRI-derived TA in our study to identify tumors and assess tumor heterogeneity in prostate cancer. Textural parameters were derived from the ROIs of mp-MRI axial slices using a first-order statistical approach among 18 patients for both tumor and non-tumor regions. Our results reveal that textural features derived from ADC maps at specific filter sizes could differentiate non-tumor from significant tumor regions in the patient population study. When maintaining the false discovery rate at 5% using the Benjamini–Hochberg correction (Appendix A, Table A1), the *p*-values for ADC skewness at a 6 × 6 px filter remained significant (*p* = 0.001). Our outcomes (ROC-AUC 0.82), with an ROC-AUC ranging from 0.73 to 0.86 in the prostate, correspond with those in the literature [15,32,33].

Skewness refers to the histogram asymmetry. Our results indicate that ADC skewness was the highest categorizing textural attribute (ROC-AUC 0.82 on ADC 6 × 6 px filter image) with a greater ROC-AUC over the textural metrics of ADC skewness with 3 × 3 and 9 × 9 px filter images. Furthermore, the skewness of the ADC image using a 9 × 9 px filter was the second-highest textural feature (ROC-AUC = 0.66) and was not statistically significant (*p* = 0.08). We anticipate that textural metrics, dependent on the relationship between pixels in a specific area of interest instead of actual pixel intensities, will have greater performance in accurately determining changes across individuals and scanners [15]. In their work, Donati et al. [34] stated that in cancers densely populated with tumor tissue, the generated ADC distribution reduces skew and spread. As a result, the variations between the analyzed ADC metrics may be reduced in these lesions. The skewness distribution, similar to cancer heterogeneity, is evaluated using the respective variable from pathologic examination, including a quantifiable measure evaluating the concentration of cancer tissues instead of the Gleason score [34]. Another outcome was that skewness is estimated through T_1_- and T_2_-weighted images for all filters, under-detecting the prevalence of prostate cancer in this study.

The histogram kurtosis reflects the peakedness of a histogram, which is associated with immature vascularity in tumor sites [35]. Entropy is a descriptor parameter that measures disorder in the distribution of signal intensities. In this group, the kurtosis and entropy of ADC and T_1_- and T_2_-weighted images for all filter scales were not significantly connected and were therefore able to allow discerning between healthy and lesion regions. These results are consistent with the literature; Bates et al. also stated that they could not find significant variations in kurtosis and entropy across regions with or without tumors using mp-MRI at 3 T [36]. After evaluating kurtosis and entropy for all pixels within the ROIs, both with and without tumors, the portion of tumor pixels may not have substantially affected kurtosis and entropy. Nevertheless, Sidhu et al. found significant prostate cancer using mp-MRI at 1.5 T for transition zone (TZ) tumors considered using template-mapping biopsy. When the tumor was removed from the slice, kurtosis became insignificant even though we performed 3 T mp-MRI scans prior to prostatectomy, and we could not detect any statistically significant variations in kurtosis or entropy between patients with and without cancer. Sidhu et al. did not use a standardized magnetic resonance imaging acquisition technique.

Although all patients had their prostate tissue histopathologically examined, every patient identified with prostate tumor tissue showed significant PSA levels in the TZ or PZ. Due to the use of 3 T scans before prostatectomy, the probability was minimal that tissue sampling would provide even limited information about the tumor’s exact position inside the prostate organ, and no sign of its positioning in any precise prostatic region [36].

A few limitations were identified in this study. First, this study may have been limited by the population size, since there were only 18 participants. However, these data are not publicly available, and patient consent must be provided at a sensitive point in their care. This study was cross-sectional, demonstrating the sensitivity of the textural analysis to detect pathology depending on the investigated time points. However, it would be interesting to perform a larger study to determine how early this technique can detect pathology. Furthermore, the filtration technique used in this study was a fast Fourier transform-based bandpass filter analysis, whereas Bates et al. used a Laplacian-of-Gaussian bandpass filter. Thus, they could not discriminate between the occurrence or absence of a tumor based on either kurtosis or entropy. Second, the study was limited by the short time interval from biopsy to mp-MRI. The time interval from biopsy to mp-MRI was a maximum of one month in this study, relatively shorter than is recommended [37]. Nevertheless, current guidelines suggest not postponing mp-MRI after biopsy for tumor identification. However, hemorrhage and inflammation after a biopsy can influence prostate MRI staging. For staging, a 6-week or longer gap between biopsy and MRI is recommended [37].

This study revealed certain significant aspects. The use of prostatectomy specimens with tumor mapping is an important distinguishing feature of current histological research. Histopathological data were used to correlate mp-MRI imaging features and a precise pathology instead of the relationship being assumed from a representative biopsy. The outcomes associated with prior studies endorse the use of mp-MRI TA in diagnosing prostate tumors. Our determinations also corroborate the use of mp-MRI TA by assisting in the identification of malignant prostate cancer.

## 5. Conclusions

Our research demonstrates that mp-MRI TA is a non-invasive approach suitable for appraising tumor heterogeneity. This procedure may help avoid excessive misdiagnosis and the overtreatment of any clinically negligible disease. Our study suggests that the skewness of ADC MRI is a significant textural parameter. We differentiated significant tumor and non-tumor regions despite a small N number and with strict inclusion criteria based on the time between scans and histology.

Our future plan is to apply these techniques to a larger population dataset and identify pathology using a machine learning approach. Another prospective application is an mp-MRI computer-aided detection (CAD) method, for which a textural measure may enhance prostate tumor classification specifically in situations where radiologists are unsure.

## Figures and Tables

**Table 1:** The demographic data and ROIs for significant tumors.

Tumor Significance	Subject Number	Mean Years	Mean PSA (ng/mL)	Mean Area (cm^2^)
Significant (≥3 + 4)	18	63	7.5	1.58

**Table 2:** Multiparametric MRI sequence parameters used in this study, including T_1_, T_2_, and ADC map images.

Sequence Parameter	T_1_	T_2_	ADC Map Image
Repetition time (ms)	4755.32	724.99	4137.73
Echo time (ms)	100	6.48	84.94
Flip angle (degrees)	90	90	90
Bandwidth (Hz/px)	210	620	2068
Field of view (mm)	180	180	180
Phase FoV %	100	100	100
Slice thickness (mm)	3	3	4
Slice gap (mm)	3	3	4
Average	2	1	7
Phase encoding direction	COL	ROW	COL
Base matrix	256	560	144
Number of acquisitions	5	7	15
Acquisition duration(s)	217.49	304.34	297.91

**Table 3:** Illustrating mean ± SEM value of the ADC map and T_2_- and T_1_-weighted images from a first-order statistical analysis of skewness for ROIs containing non-tumors and significant tumors. *p*-values for differences between ROIs were computed using two-tailed Mann–Whitney U testing. The comparison between ROIs, including tumors and non-tumors, was assessed according to the area under the receiver operator characteristic curve (ROC-AUC) for mp-MRI TA parameters.

Sequence	Non-Tumor Regions (Mean ± SEM)	Significant Tumor (Mean ± SEM)	*p*-Value	ROC-AUC (95% CI)
ADC 3 × 3	0.06 ± 0.12	0.43 ± 0.15	0.94	0.66
T2 3 × 3	0.28 ± 0.94	0.01 ± 0.10	0.54	0.31
T1 3 × 3	−0.16 ± 0.78	0.12 ± 0.10	0.29	0.39
ADC 6 × 6	−0.14 ± 0.13	0.49 ± 0.11	0.001	0.82
T26 × 6	0.14 ± 0.15	0.06 ± 0.03	0.8	0.47
T1 6 × 6	0.05 ± 0.13	0.06 ± 0.17	0.2	0.37
ADC 9 × 9	−0.13 ± 0.12	0.17 ± 0.12	0.08	0.66
T2 9 × 9	−0.03 ± 0.16	0.12 ± 0.10	0.48	0.43
T1 9 × 9	−0.20 ± 0.17	0.16 ± 0.18	0.5	0.56

**Table 4:** Illustrating mean ± SEM value of the ADC map and T_2_- and T_1_-weighted images acquired from a first-order statistical analysis of kurtosis for ROIs containing non-tumors and significant tumors. *p*-values of differences between ROIs were computed using two-tailed Mann–Whitney U testing. The comparison between ROIs, including tumors and non-tumors, was assessed according to the area under the receiver operator characteristic curve (ROC-AUC) for mp-MRI TA parameters.

Sequence	Non-Tumor Regions (Mean ± SEM)	Significant Tumor (Mean ± SEM)	*p*-Value	ROC-AUC (95% CI)
ADC 3 × 3	−0.72 ± 0.10	−0.41 ± 0.22	0.37	0.58
T2 3 × 3	0.13 ± 0.11	0.19 ± 0.15	0.6	0.55
T1 3 × 3	−0.57 ± 0.14	0.37 ± 0.40	0.46	0.57
ADC 6 × 6	−0.64 ± 0.16	−0.47 ± 0.19	0.22	0.61
T2 6 × 6	−0.01 ± 0.24	−0.34 ± 0.16	0.72	0.53
T1 6 × 6	0.01 ± 0.19	0.22 ± 0.43	0.65	0.54
ADC 9 × 9	−0.78 ± 0.08	−0.65 ± 0.10	0.81	0.52
T2 9 × 9	−0.09 ± 0.19	−0.51 ± 0.14	0.82	0.47
T1 9 × 9	−0.08 ± −0.29	−0.05 ± 0.45	0.56	0.55

**Table 5:** Illustrating mean ± SEM value of the ADC map and T_2_- and T_1_-weighted images acquired from a first-order statistical analysis of entropy for ROIs containing non-tumors and significant tumors. *p*-values of differences between ROIs were computed using two-tailed Mann–Whitney U testing. The comparison between ROIs, including tumors and non-tumors, was assessed according to the area under the receiver operator characteristic curve (ROC-AUC) for mp-MRI TA parameters.

Sequence	Non-Tumor Regions (Mean ± SEM)	Significant Tumor (Mean ± SEM)	*p*-Value	ROC-AUC (95% CI)
ADC 3 × 3	4.09 ± 0.09	4.07 ± 0.15	0.84	0.48
T2 3 × 3	6.25 ± 0.11	6.10 ± 0.22	0.75	0.46
T1 3 × 3	5.61 ± 0.30	5.48 ± 0.31	0.71	0.53
ADC 6 × 6	4.08 ± 0.09	4.15 ± 0.11	0.64	0.54
T2 6 × 6	5.92 ± 0.12	5.86 ± 0.12	0.87	0.51
T1 6 × 6	4.98 ± 0.31	4.85 ± 0.27	0.68	0.54
ADC 9 × 9	4.02 ± 0.10	4.12 ± 0.11	0.54	0.55
T2 9 × 9	5.72 ± 0.13	5.59 ± 0.16	0.56	0.55
T1 9 × 9	4.58 ± 0.27	4.60 ± 0.24	0.89	0.48

## Data Availability

The data and code presented in this study are available upon request from the corresponding author. The data are not openly accessible because of privacy constraints.

## Appendix A

**Table A1:** Benjamini–Hochberg approach for false discovery rate (FDR) of *p*-values for all patients.

Ranking	*p*-Value	Adjusted *p*-Value	Rejected
1	0.001	0.001852	1
2	0.08000	0.003704	0
3	0.20000	0.005556	0
4	0.22000	0.007407	0
5	0.29000	0.009259	0
6	0.37000	0.011111	0
7	0.46000	0.012963	0
8	0.48000	0.014815	0
9	0.50000	0.016667	0
10	0.54000	0.018519	0
11	0.54000	0.020370	0
12	0.56000	0.022222	0
13	0.56000	0.024074	0
14	0.60000	0.025926	0
15	0.64000	0.027778	0
16	0.65000	0.029630	0
17	0.68000	0.031481	0
18	0.71000	0.033333	0
19	0.72000	0.035185	0
20	0.75000	0.037037	0
21	0.80000	0.038889	0
22	0.81000	0.040741	0
23	0.82000	0.042593	0
24	0.84000	0.044444	0
25	0.87000	0.046296	0
26	0.89000	0.048148	0
27	0.94000	0.050000	0
